# NOTCH1 inhibition enhances the efficacy of conventional chemotherapeutic agents by targeting head neck cancer stem cell

**DOI:** 10.1038/srep24704

**Published:** 2016-04-25

**Authors:** Zhi-Li Zhao, Lu Zhang, Cong-Fa Huang, Si-Rui Ma, Lin-Lin Bu, Jian-Feng Liu, Guang-Tao Yu, Bing Liu, J. Silvio Gutkind, Ashok B. Kulkarni, Wen-Feng Zhang, Zhi-Jun Sun

**Affiliations:** 1 The State Key Laboratory Breeding Base of Basic Science of Stomatology & Key Laboratory of Oral Biomedicine, Ministry of Education, Wuhan, China; 2 Department of Oral Maxillofacial-Head Neck Oncology, School and Hospital of Stomatology, Wuhan University, Wuhan, China; 3 Oral and Pharyngeal Cancer Branch, National Institute of Dental and Craniofacial Research, National Institutes of Health, Bethesda, MD USA; 4 Functional Genomics Section, Laboratory of Cell and Developmental Biology, National Institute of Dental and Craniofacial Research, National Institutes of Health, Bethesda, MD USA

## Abstract

Cancer stem cells (CSCs) are considered responsible for tumor initiation and chemoresistance. This study was aimed to investigate the possibility of targeting head neck squamous cell carcinoma (HNSCC) by NOTCH1 pathway inhibition and explore the synergistic effect of combining NOTCH inhibition with conventional chemotherapy. NOTCH1/HES1 elevation was found in human HNSCC, especially in tissue post chemotherapy and lymph node metastasis, which is correlated with CSCs markers. NOTCH1 inhibitor DAPT (GSI-IX) significantly reduces CSCs population and tumor self-renewal ability *in vitro* and *in vivo*. Flow cytometry analysis showed that NOTCH1 inhibition reduces CSCs frequency either alone or in combination with chemotherapeutic agents, namely, cisplatin, docetaxel, and 5-fluorouracil. The combined strategy of NOTCH1 blockade and chemotherapy synergistically attenuated chemotherapy-enriched CSC population, promising a potential therapeutic exploitation in future clinical trial.

Accumulating evidence indicated that tumors are frequently composed of heterogeneous cell types and that tumor initiation and growth are driven by a subpopulation of cells, termed cancer stem cells (CSCs) or tumor-initiating cells^1^. CSCs share certain properties with normal stem and/or progenitor cells. They have accumulated oncogenic mutations and lost normal constraints on growth control, and can be preferentially resistant to chemo-radiotherapy^2^,^3^. Cancer treatment that fails to eliminate CSCs may allow relapse and even diaspora of the original tumor^2^,^3^. Thus, developing corresponding specific strategies that target CSCs for a better cure is essential.

Despite advances in diagnosis and treatment, head neck squamous cell carcinoma (HNSCC) remains one of the most prevalent types of malignancy worldwide, with an incidence of more than 500,000 cases every year^4^,^5^. The mortality related to HNSCC is mainly caused by local recurrence and local metastasis to cervical lymph node and occasionally by distant organ metastasis^4^,^5^. The five-year survival rate of patients with advanced HNSCC has only marginally improved over the past three decades with even poorer prognosis in oral cavity (approximately 90% of HNSCC)^6^. Ongoing efforts aimed at elucidating the deregulation of molecules that contribute to HNSCC stemness maintenance may, to some extent, help increase the overall survival of surgical and radio-chemotherapeutic interventions^6^. A recent study reported the existence of CSCs or cancer stem-like cells that facilitate tumor relapse, which demonstrates the importance to understand why HNSCC does not respond to chemotherapy and to identify new targeted treatments^7^.

NOTCH signaling plays a fundamental role in the implementation of differentiation, proliferation, and self-renewal in disease development^8^. Accumulated evidence suggests that aberrant NOTCH1 signaling has been implicated in a variety of tumors^9^,^10^,^11^, especially in HNSCC, which surprisingly comprises about 50% of activated mutation in Chinese population^12^,^13^ and 10% in Caucasian population^14^,^15^. In terms of CSC maintenance, NOTCH pathway participates in epithelial–mesenchymal transition and increases chemoresistance^16^. Previous studies have indicated that inhibition of NOTCH1 exploiting monoclonal antibody or pathway inhibitor resulted in broad-spectrum antitumor activity in cancer cell line-based xenograft models^17^, which contributed to CSC inhibition through chemotherapeutic treatment^18^,^19^,^20^.

In this study, we confirmed that NOTCH1 inhibition delays tumorigenesis and effectively reduces CSC self-renewal of nude mice HNSCC xenograft. Furthermore, chemotherapeutically combined NOTCH1 inhibitor synergistically attenuated chemotherapy-enriched CSC population *in vitro* and *in vivo*, which provides the possibility to effectively eliminate head and neck CSCs.

## Results

### Elevated NOTCH1/HES1 signaling activity in human HNSCC is associated with CSCs

**Figure 1:**
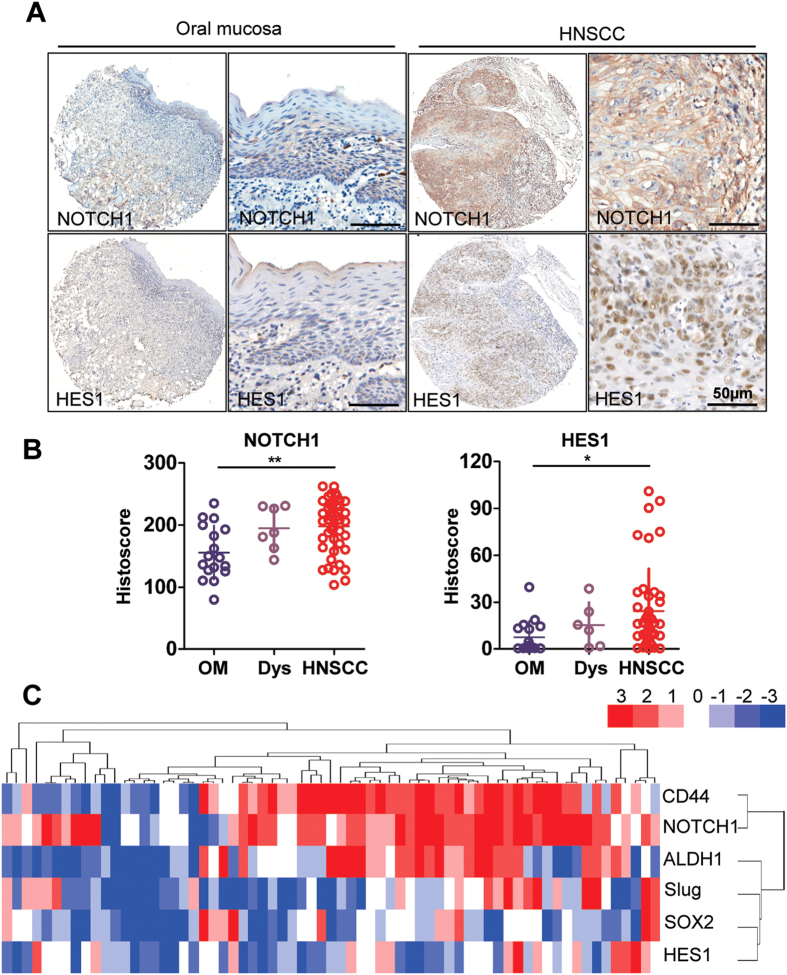
NOTCH signaling is activated in human HNSCC. (**A**) Over-expression of NOTCH1, HES1 inhuman HNSCC (right panel) as compared with normal oral mucosa (OM, left panel) and mucosal dysplasia (Dys); scale bars, 50 μm. (**B**) Histoscore of immunohistochemical staining of NOTCH1 (left panel) and HES1 (right panel). Each sample score present as a dot, One way ANOVA followed by Tukey test by Graph Pad Prism 5, *P < 0.05; **P < 0.01. (**C**) Hierarchical clustering of NOTCH1, HES1 with CD44, SOX2, ALDH1 and Slug. Immunohistochemical staining was clustered with Cluster and visualized with Java Treeview.

Previous whole exome sequencing reports have shown that *NOTCH1* gene mutations^12^,^13^,^14^,^15^,^21^ were present in about 10% to 50% of HNSCCs, whereas the exact role of NOTCH signaling in HNSCC is still unclear. To target NOTCH pathway in human HNSCC, we searched The Cancer Genome Atlas (TCGA) dataset^21^ and ONCOMINE database^22^. Data retrieved from TCGA head neck cancer dataset suggested that DNA copy number of *HES1*, a putative downstream target of *NOTCH1*, significantly increased in human HNSCC than in its control counterpart (*P* = 1.06E-51,Fig. S1A). Dataset from Peng^23^ confirmed that the mRNA level of head neck cancer is significantly higher compared with that of oral mucosa (*P* = 1.156E-18, Fig. S1B). To determine the specific expression of NOTCH1 pathway, we first validated the protein levels in HNSCC cell lines FaDu, CAL-27, and UM-SCC-23 and compared them with primary cultured normal oral squamous epithelia keratinocyte (OKC). As shown in Fig. S1C, the protein levels of NOTCH1 and HES1 were strongly expressed in FaDu and fairly strong in all three HNSCC cell lines compared with OKC. This observation was validated by the mRNA level of *NOTCH1* and *HES1* in HNSCC cell lines through qPCR (Fig. S1D). To further observe the protein expression of NOTCH1 in HNSCC, we took advantage of human HNSCC tissue microarray. As expected, NOTCH1 (*P* < 0.01) and HES1 (*P* < 0.05) were over expressed in HNSCC (*n* = 59) than in normal oral mucosa samples (*n* = 39, Fig. 1A,B). Elevated NOTCH1 protein was mainly located in the carcinoma nest area and middle differentiation districts in tumor sample, whereas low expression was observed in the basal layer or sporadically distributed in reticular layer (Fig. 1A). HES1 was almost negative in the normal mucosa, whereas extensive expression was observed in the nucleus (Fig. 1A).

We further investigated the correlation between NOTCH1 signaling and CSCs. We first examined the expression of literature-reported HNSCC CSC self-renewal-related markers, namely, CD44, SOX2, ALDH1, and Slug. Interestingly, all these self-renewal markers showed increased levels in HNSCC tissue (Fig. S2A). The expression of NOTCH1 (CD44, r = 0.92; ALDH1, r = 0.48; SOX2, r = 0.94; Slug, r = 0.29, Fig. S2B) and HES1 (CD44, r = 0.72; ALDH1, r = 0.42; SOX2, r = 0.91; Slug, r = 0.37, Fig. S2C) significantly correlated with CSC markers. To better visualize the correlation between NOTCH1 signaling and CSC markers, we conducted hierarchical cluster analysis, which showed that selectively high NOTCH1 expression may interact more intimately with CD44, whereas HES1 correlated with SOX2, Slug, and ALDH1 more closely (Fig. 1C). Therefore, a universal abnormal over-expression of NOTCH1 signaling pathway is presented, and NOTCH1 signaling is closely correlated with CSC self-renewal markers, which indicates potential roles in CSC regulation.

**Figure 2:**
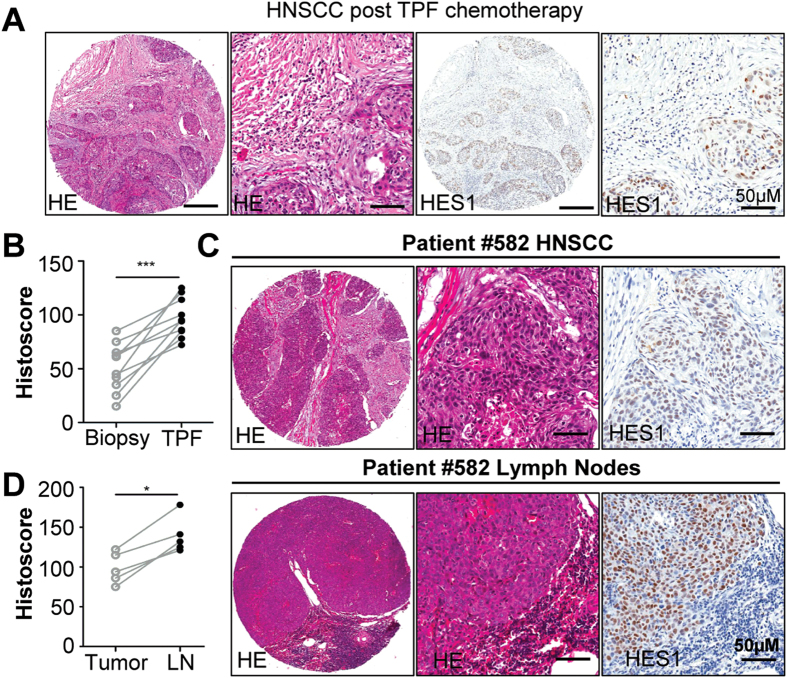
Increase expression of HES1 in cisplatin based chemotherapeautic HNSCC. (**A**) Representative hematoxylin-eosin and immunohistochemical staining of HES1in HNSCC patient receiving TPF neoadjuvant chemotherapy with quantitative histoscore in (**B**) Scale bar, 50 μm. (**C**) Representative immunohistochemical staining of HES1 with quantitative histoscore Representative hematoxylin-eosin and immunohistochemical staining of HES1in HNSCC patient receiving TPF neoadjuvant chemotherapy with quantitative histoscore in (**D**). Each sample score present as a dot, paired *t* test, *P < 0.05; ***P < 0.001.

To determine the role of NOTCH1 pathway in chemotherapy, we utilized cherish paired specimen (postchemotherapeutic specimen and prechemotherapeutic biopsy in the same patient) with induction-combined TPF chemotherapy of human HNSCC using docetaxel (DTX), cisplatin (CDDP) and 5-fluorouracil (5-FU) for validation^24^. In deed, significant increase of HES1 expression level was observed in post-TPF chemotherapy compared with biopsy specimen (Fig. 2A,B), which suggests that increased NOTCH1 pathway may be correlated with chemoresistance. Interestingly, we also found immunoreactivity of HES1 in metastatic lymph nodes (Fig. 2C,D).

### NOTCH1 signaling blockade attenuates CSC phenotype in HNSCC *in vitro*

**Figure 3:**
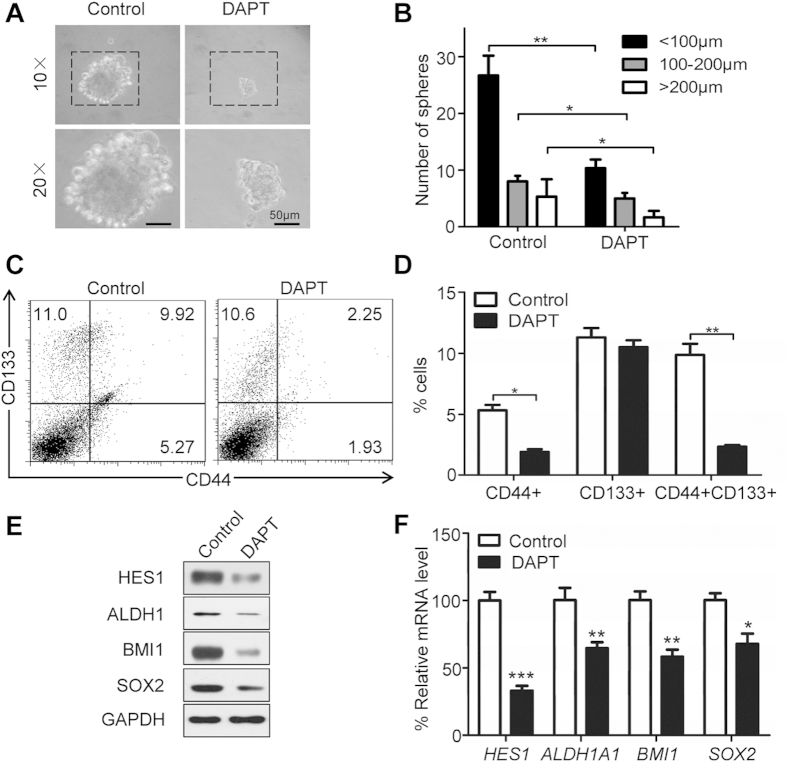
NOTCH inhibition attenuates cancer stem cells phenotype of HNSCC in CAL27 cell line. (**A**) Representative images of CAL27 *in vitro* tumor sphere formation assay after 14days culture in the serum free media with or without 10 μM DAPT; Scale bar, 50 μm. Experiment was repeated twice in triplicate. (**B**) Quantification of the number and size of tumor spheres with or without DAPT treatment; Experiment repeated twice and data presented as mean ± SEM, *P < 0.05. (**C**) Representative flow cytometry and quantification (**D**) indicate decrease CD44+ CD133+ and CD44+ CD133− cell population by DAPT treatment; Experiment was repeated twice. *P < 0.05; **P < 0.01. (**E**) Western blotting analysis shows DAPT decrease HES1, ALDH1, BMI1 and SOX2 expression in HNSCC CAL27 cell line. (**F**) The mRNA levels of *HES1, ALDH1A1*, *BMI1* and *SOX2* were analyzed with qPCR; *P < 0.05; **P < 0.01.

To determine whether NOTCH pathway activity is required for the survival or self-renewal of tumor sphere-forming cells, we used DAPT, a novel γ-secretase inhibitor, which is required for prophylactic processing of NOTCH1 receptors. To confirm the on-target effect of NOTCH signaling inhibition by DAPT, we test 5 μM and 10 μM DAPT with 2 strand of NOTCH1 siRNA. As shown in Fig. S3A both siRNA may reduce NOTCH1 and HES1 after 24 h transfection, and both 5 μM and 10 μM DAPT may decrease NOTCH1 and HES1 expression after 24 h treatment in human HNSCC cell line CAL27 and FaDu. Interestingly, we found that CAL27 and FaDu HNSCC cells in HNSCC formed tumor spheres that are directly proportional to the number of cells seeded. As shown in Figs 3A and S4A, NOTCH signaling blockade with DAPT could fairly reduce the size of tumor spheres, indicating self-renewal or initiation ability, compared with vehicle control. Moreover, the number of tumor-spheres for NOTCH1 blockade showed significant decrease compared with the control group, regardless of size distribution (Figs 3B and S4B). The effect of reduced tumor sphere may not due to the inhibition of cell viability as indicated by unchanged cell number of 5 μM and 10 μM DAPT treatment (Fig. S3B) in CAL27 and FaDu cell lines. To better understand whether NOTCH1 signaling blockade reduces CSC phenotype, we used flow cytometry to assess CSC proportion with CD44 and CD133 as specific CSC surface markers in HNSCC^25^. As shown in Fig. 3C,D, NOTCH1 blockade can significantly decrease the CSC percentage of CD4+ CD133+ (*P* < 0.01) and CD44+ cells (*P* < 0.05) in HNSCC, whereas no statistical significance was observed for CD133+ cells. This experiment is repeatable in FaDu cell line (Fig. S4).To further confirm the effect of NOTCH1 blockade, we found the reduced protein expression (Figs 3E and S4E) as well as mRNA levels of HES1 (Figs 3F and S4F), a direct target of NOTCH1 signaling. Remarkably, self-renewal marker ALDH1, BMI1 and SOX2 protein level are decreased as indicated by Western blot (Figs 3E and S4E) and qPCR (Figs 3F and S4F) in CAL27 and FaDu cell lines. The results suggest that NOTCH1 blockade could not only decrease CSC proportion but also reduce CSC-related marker and self-renewal transcription factors from the mRNA and protein levels, indicating that NOTCH blockade can truly inhibit CSC phenotype with DAPT.

### NOTCH1 blockade reduces CSC phenotype in HNSCC xenograft mouse model

**Figure 4:**
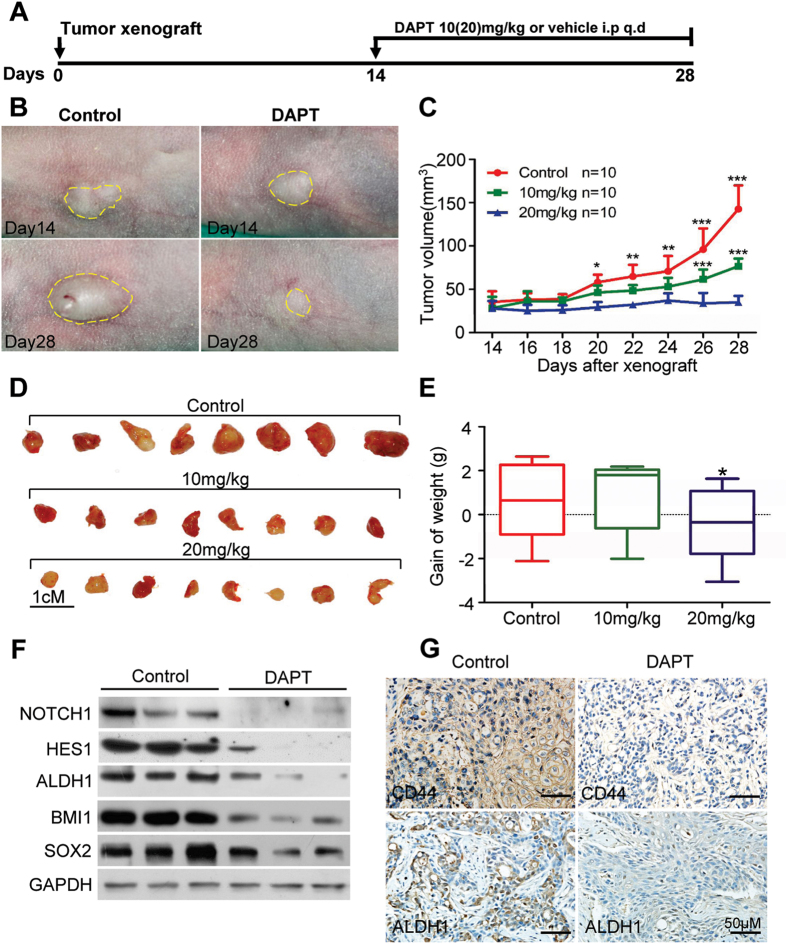
NOTCH1 inhibition reduces stemloid cancer cell in CAL27 xenograft model. (**A**) Schematic diagram for the CAL27 xenograft implantation and drug delivery. NOTCH signaling inhibitor DAPT (10 mg/kg, 20 mg/kg, respectively) or equivalent volume 5% ethanol PBS (vehicle) was given by intraperitoneal injection (i.p) once a day (q.d) in CAL27 cells xenograft nude mice for consecutively two weeks (*n* = 10 mice, respectively). (**B**) Representative images showed tumor regression in HNSCC xenograft treated with DAPT (right) as compared with control group. Dahs lines were utilized to depict the outline of tumor lump. Scale bar, 1 cM. (**C**) 10 mg/kg and 20 mg/kg DAPT treatment significant delay tumor growth; mean ± SEM, *P < 0.05; **P < 0.01; ***P < 0.001. (**D**) Tumor lump dissected from xenograft mice at 28 days after euthanasia; scale bar, 1 cm. (**E**) Box and whiskers plot revealed the weight changes of DAPT- or vehicle-treated mice; *P < 0.05, one-way ANOVA. (**F**) Western blotting analysis indicated DAPT treatment decreased cancer stem cells related markers ALDH1, BMI1 and SOX2 as compared with control group. GAPDH was used as loading control. (**G**) Representative photo of mice xenograft showed DAPT reduced CD44 and ALDH1 immunoreactivity as compared with control group; Scale bar, 50 μm.

To evaluate the activity and tolerability of NOTCH1 inhibitor DAPT in HNSCC *in vivo*, pilot dosage studies were conducted using CAL27 as human HNSCC CAL27 xenograft models. A total of 10^6^ cells/mouse CAL27 cells were implanted subcutaneously into the flank of nude mice, and treatment was initiated beginning 14 days after tumor inoculation. As shown in Fig. 3A, 10 and 20 mg/kg of DAPT or vehicle were given intraperitoneally (i.p.) every day for 14 consecutive days (Fig. 4A). As shown in Fig. 4B, tumor volume was remarkably reduced after two-week DAPT treatment compared with control group. Furthermore, both 10 and 20 mg/kg DAPT could significantly reduce tumor growth after two-week treatment (Fig. 4B–D, *n* = 10, respectively; *P* < 0.001). Considering the digestive side effect of NOTCH1 blockade, we evaluated the toxicity of different dosages with body weight changes, in which no statistical significance between control and 10 mg/kg group was presented. On the contrary, the 20 mg/kg group showed obvious weight reduction than the control (Fig. 4E, *P* < 0.05), indicating that 20 mg/kg may be toxic to nude mice. Therefore, we chose 10 mg/kg as the appropriate dosage for the following animal study. To confirm whether the tumor decrease was directly correlated with CSC blockade, we compared the molecular expression of NOTCH1 and HES1 in 10 mg/kg or vehicle group xenograft mice using Western blot and immunohistochemistry. Indeed, DAPT could decrease NOTCH1 and HES1 (Fig. 4F), which indicates that the decrease of tumor size in nude mice by DAPT is an on-target effect. Self renewal marker ALDH1, BMI1 and SOX2 were found to be reduced after DAPT treatment through Western blot (Fig. 4F). CSC marker CD44 and ALDH1 decreased in DAPT treatment group after immunostaining (Fig. 4G). Therefore, it indicated that NOTCH1 blockade could decrease tumor growth through target CSC phenotype in HNSCC xenograft mouse model.

### NOTCH1 inhibition attenuates chemoreagent-enriched HNSCC CSC population *in vitro* and *in vivo*

**Figure 5:**
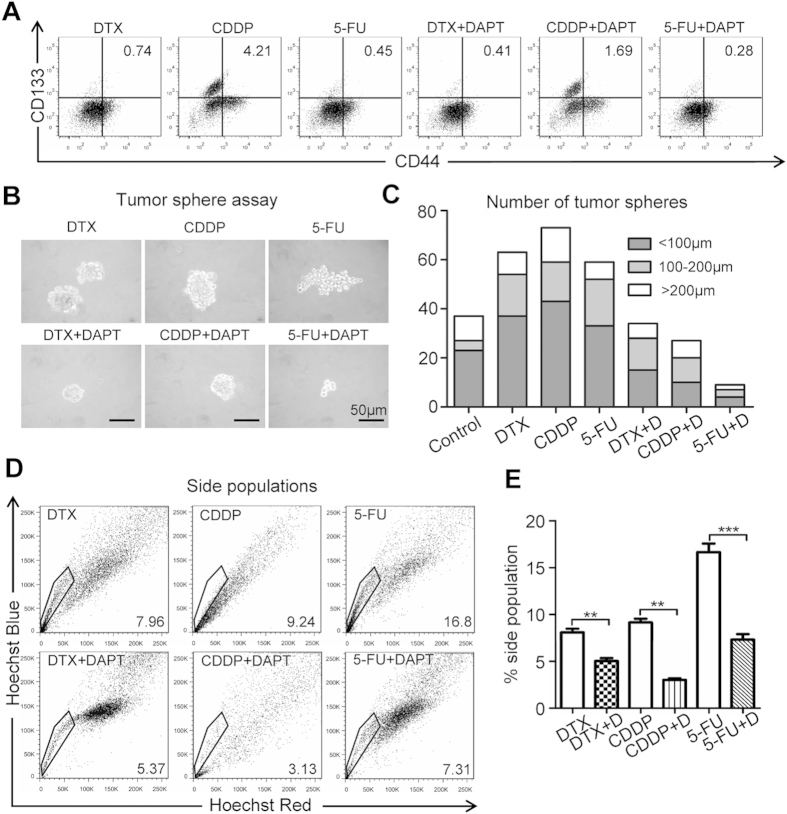
Suppression of NOTCH signaling inhibits chemoresistance in CAL27 cell line. (**A**) Representative flow cytometry photos shows DAPT treatment attenuate chemotherapy agent Docetaxel (DTX, 10 μM), cisplatin (CDDP, 10 μM), 5-fluorouracil (5-FU, 15 μM) induced CD44+ CD133+ CAL27 cell population. Experiment repeated twice. (**B**) Representative tumor sphere formation of combined DAPT treatment with chemotherapeutic agents; Scale bar, 50 μm. (**C**) Quantification of *in vitro* sphere number and size of DTX, CDDP, 5-FU treated CAL27 with or without DAPT. (**D**) Representative flow cytometry shows side population changes of DTX, CDDP, 5-FU treated CAL27 cells with or without DAPT. Stained with Hoechst 33342 and excited by UV light; Hoechst Red, 675 nm channel; Hoechst Blue, 450 nm channel. (**E**) Quantification of side populations from triple experiments; Data present as mean ± SEM, **P < 0.01; ***P < 0.001.

Myriad reports suggested the important function of chemoresistant and chemotherapy-enriched CSCs. We used flow cytometry to detect CSC percentage to further determine the direct role of NOTCH1 inhibition in chemoreagent-enriched CSC population. Rare CD44+ CD133+ population (0.10 ± 0.03%) in CAL27 (Fig. 5A) and FaDu (Fig. S5A) HNSCC cell line was found. Indeed, single treatment with 10uM DTX, 10 uM CDDP, or 15 uM 5-FU may enrich CD44+ CD133+ cell population. Combinatorial chemotherapy with 10 uM DAPT may reduce CD44+ CD133+ cell population (Figs 5A and S5A). To further validate the role of combination therapies on self-renewal ability, *in vitro* tumor sphere formation assay was assessed for chemotherapeutical agents or in combination with DAPT. As expected, DAPT combined with chemotherapeutical agents could decrease not only the size of tumor spheres but also the number of tumor spheres regardless of size profile of CAL27 cell line (Fig. 5B,C) as well as FaDu cell line (Fig. S5B). We then used side population discrimination assay to further analyze the effects of NOTCH1 in CSCs based on the differential potential of cells to efflux the Hoechst dye via the ATP-binding cassette family of transporter proteins expressed within the cell membrane. Consistently, DAPT combined with chemotherapeutical agents significantly decreased the side population of chemoreagent-enriched CD44+ CD133+ CAL27 cell population (DTX, *P* < 0.01; CDDP, *P* < 0.01; 5-FU*, P* < 0.001, Fig. 5D,E) and FaDu cell line (Fig. S5C and D). Collectively, the combination of NOTCH1 inhibition may reduce conventional chemoreagent-enriched CSC population *in vitro*.

**Figure 6:**
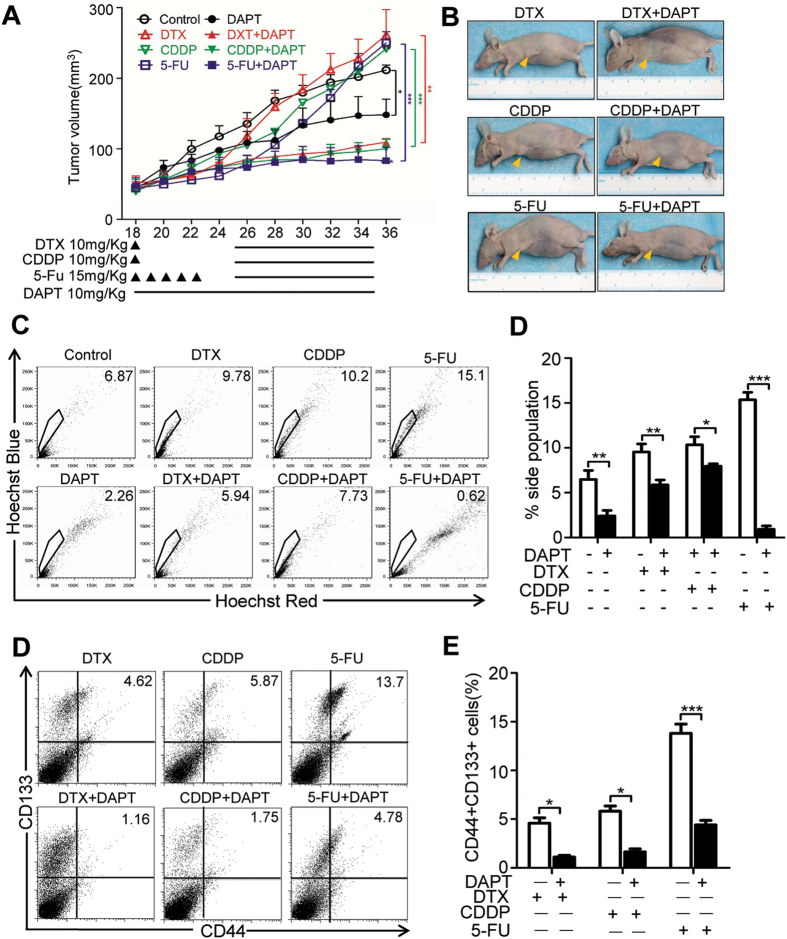
NOTCH inhibition reduces chemoresistance of CAL27 cell xenograft. (**A**) Tumor growth curve and (**B**) representative photo of DTX, CDDP, 5-FU, DAPT or combinational chemotherapy. 10 mg/kg DTX or CDDP were infused once at day 18 and 15 mg/kg 5-FU was infused for consequent 5 days from day18 to day 22. The DAPT only group receive 10 mg/kg DAPT every day and combined group receive additional 10 mg/kg DAPT every day from (as indicated by dash) from day 25 to day 35. *n* = 5 mice respectively. Data represents the mean ± SEM of tumor volumes from each group.*P < 0.05; ***P < 0.01. (**C**) DAPT effectively decreased the proportion of side population directly harvest from CAL27 xenografts tumor as compared with independent chemotherapeutical drugs. Xenograft tumor were stained with Hoechst 33342 and excited by UV light; Hoechst Red, 675 nm channel; Hoechst Blue, 450 nm channel. (**D**) Quantification of side population from triple experiments; *P < 0.05; **P < 0.01; ***P < 0.001. (E) DAPT effectively decreased the percentage of CD44+ CD133+ cell population in CAL27 xenograft. Digits indicated the percentage of CD44+ and CD133+ double positive cells. (**E**) Quantification of side population from triple experiments; **P < 0.01; ***P < 0.001.

To further determine whether NOTCH1 inhibition reduces chemoreagent-enriched CSC, we reconsidered the xenograft mice model. We started with single reagent chemotherapy of DTX, CDDP, and 5-FU. We started DAPT treatment 14 days after inoculation. As shown in Fig. 6A,B, combinatorial chemotherapy with DAPT may synergistically act to reduce tumor growth. We harvested the xenograft from mice and analyzed it through flow cytometry to confirm whether the reduction of tumor growth is an on-target effect of CSCs. As shown in Fig. 6C, combined DAPT treatment significantly reduced the side population of DTX- (*P* < 0.01), CDDP-, (*P* < 0.05) and 5-FU-enriched (*P* < 0.001) side population, which has been considered as CSC-enriched population. Consistent with the aforementioned study, DAPT (*P* < 0.01) may reduce side population compared with the control group (Fig. 6C). We also examined CD44+ CD133+ cell population in xenograft, which indicated that combined DAPT treatment reduces DTX-, CDDP-, and 5-FU-enriched CD44+ CD133+ cell population (Fig. 6D,E). Interestingly, 5-FU may significantly enrich side population and CD44+ CD133+ cell population in CAL27 xenograft, which may be effectively attenuated by combinatorial NOTCH inhibition. Therefore, combinational NOTCH1 inhibition by DAPT may significantly reduce tumor growth by reducing CSC population in HNSCC *in vitro* and *in vivo*.

## Discussion

Oncology is witnessing a dramatic increase in novel therapies, but the steps forward have been tenuous^26^. Notably, the most effective targeted therapies may have activity against CSCs or the stem cell niche^18^. The present findings demonstrate that blocking NOTCH1 signaling is a promising strategy to target CSCs in HNSCC. This study confirmed, for the first time, that NOTCH1 inhibition delays tumorigenesis and effectively reduces CSC self-renewal of HNSCC. Moreover, we observed that chemotherapeutical agents combined with NOTCH1 inhibitor synergistically attenuate chemotherapy-enriched CSC population *in vitro* and *in vivo.* These data provide the possibility to efficiently eliminate bulk cell populations and cancer stem-like cells in HNSCC based on further understanding of CSCs and pharmacologic strategy targeting relevant molecular events.

*NOTCH1* mutation was extensively discovered in head and neck cancer by next-generation sequencing and high throughput gene profiling range from approximately 10% of Caucasian and above 50% of Chinese population^12^,^13^,^14^,^15^,^21^,^27^. Although detailed causes for the higher mutation rate in the Chinese population remains to be determined, differences in the genetic background of race/ethnicity and etiologic factors of HNSCC, such as high-concentration liquor and concurrent intake of tobacco and alcohol or areca, should be considered. With the established gain-of-function mutations of *NOTCH1* in T-cell acute lymphoblastic leukemia^28^, HNSCC mutation highlighted the dichotomous role constituted with either loss or gain-of-function mutations^12^,^13^,^14^,^15^,^21^,^27^. The discrepancy in the potential role of *NOTCH1* mutations may rely on the different mutation spectra in different cohort studies or population. Cohort studies of the vast majority of Caucasian mutations clustered around the “ligand-binding” domain, indicating that averting NOTCH1–ligand interaction may be the most prevalent cause of *NOTCH1*-related oral tumorigenesis^14^,^15^. The next most mutated area was the RAM/ANK region of the NICD, which is a core component of *NOTCH1* nuclear complexes that may disrupt proper nuclear complexes assembly and subsequently prevent transcription of NOTCH1-dependent genes^14^,^15^,^27^. By contrast, Asian studies revealed that more than a third of mutations are located within the EGF-like repeats, particularly around the Abruptex regions^12^,^13^. Although little is known about the contribution of EGF repeats to *NOTCH1* function, the integrity of the Abruptex (EGF repeats 24–29) is required for suppression of NOTCH1 activity, and mutations within this region enhance NOTCH1 signaling^29^. Another frequently mutated area in Asian HNSCC is membrane-proximal NRR^12^,^13^, which acts as a receptor activation switch that can lead to ligand-independent activity and are considered one of the gain-of-function-mutated regions in T-cell acute lymphoblastic leukemia^30^. Therefore, although some clustering overlaps between Caucasian and Asian tumors, the overall spectrum of *NOTCH1* mutations is profoundly different between these cohorts and therein lies the disparate role of NOTCH1 mutation in HNSCC. Despite the distinct property of *NOTCH1* mutation, activation of NOTCH1 pathway was still observed in the Caucasian population^31^. Furthermore, a cohort study of Song *et al.*^13^ revealed the relationship between NOTCH1 mutation and poor prognosis in HNSCC, which implicates the crucial role of NOTCH1 signaling in the development of HNSCC. Two direct downstream targets HES1 and HEY1 were over expressed in HNSCC^31^. The present study demonstrates that NOTCH1 signaling status is perhaps mostly gain-of-function and is highly activated in Asian population.

Recent evidence supports NOTCH1 signaling in the function of CSCs that has been thought to be responsible for the relapse and metastasis of cancer and can be regarded as a promising target for cancer therapy^18^. This study showed direct evidence that aberrantly activated NOTCH1 signaling drives CSC phenotype in human HNSCC. Hierarchical Cluster data suggests a much more complex situation of NOTCH1 and HES1 expression correlated with cancer stem cell marker CD44, ALDH1, Slug and SOX2. By using HNSCC xenograft model, we further demonstrated that inhibition of NOTCH1 signaling by γ-secretase inhibitor (GSIs) DAPT is efficacious in downsizing tumor growth and reducing CSCs in HNSCC. While, we should keep in mind that DAPT may have a wide range of targets in addition to NOTCH protein^32^. One huge challenge for CSC research is how to precisely define the CSC population. Mikhail *et al.*^33^ suggested that it is paradoxical that CSCs must be resting to explain their resistance to therapy, yet must be cycling to explain their persistence in cell culture; thus, proliferating self-renewing cells or cancer stemloids were the ones defined as CSCs in this study. Also, we need to point out that not all the sphere arises from CD44+ CD133+ cell population in this study. Recent report suggested that about 83% tumor sphere-forming cancer stem cells harvested from patient tumors are ALDH1+ cells^34^. A disadvantage of these conventional cell line-based xenograft models is that their lack of the cellular heterogeneity, and tumor-stroma interactions^35^. An disadvantage of these conventional cell line-based xenograft models is that they lack of the cellular heterogeneity, especially cross talk of cancer cell and stroma of original tumor^35^. Importantly, the effect of DAPT in HNSCC tumorigenesis may be a comprehensive effect of angiogenesis, immune cell, and CSC. Remarkably, a recent report indicated that angiogenic genes eg. NOTCH1 are important characteristic molecular signature of HNSCC CSCs^36^ and immune cells such as immature myeloid cells play important role in maintain cancer cell stemness^37^. Targeting NOTCH1 in HNSCC may simultaneously reduce both CSCs and tumor neo-angiogenesis^38^. Interestingly, the effect of DAPT on CAL27 and FaDu was not through inhibition of cell viability, which is consistent with Su’s observation in breast cancer cell lines^39^. Recent report suggested that inhibition of NOTCH signaling may promote cancer stem cell differentiation^26^. In the future, a patient derived xenograft (PDX)^40^ or genetically defined spontaneous mice model may needed to unearth the role of tumor environment on cancer stem cell.

Conventional chemotherapy that may enrich CSCs is a difficult problem. The present data are consistent with a recent study, in which CSC proportion is significantly increased in breast cancer specimens treated with neoadjuvant chemotherapy^41^. Further analysis revealed NOTCH1 signaling over-expression in post-TPF chemotherapy patients, suggesting that targeting NOTCH1 may render wonderful opportunity to decrease chemotherapy-enriched CSCs. Although molecular therapies targeting CSCs show great promise, majority of targeted therapies may be mostly effective when used in conjunction with cytotoxic therapies or other targeted therapies, such as chemotherapy^42^. Thus, the apparent resistance of CSCs to conventional therapies does not mean that these therapies will not remain to be the mainstays of cancer treatment^33^. Rather, the greatest improvement in tumor control will likely initially be through the identification of resistance mechanisms within CSCs that may be disrupted to augment durability of patient response. Indeed, cytotoxic reagent CDDP not only enrich HNSCC CSCs by debulk proliferating cancer cell but also enhance stem cell fraction by induce putative self renewal marker Bmi1^43^. As mentioned above, NOTCH1 signaling inhibition could not only disrupt the maintenance of CSCs but also reduce chemoresistance of CSCs triggered by CDDP, DOX, and 5-FU. The targeted depletion of stem-like cells following conjunction with NOTCH pathway blockade sharply differs with prior reports of CSC survival following standard chemotherapies^2^. In support of this concept, a flurry of subsequent studies that aimed to explore avenues for more specific NOTCH1 targeting has been conducted^44^. Combination of NOTCH1 blockade and chemotherapeutic oxaliplatin resulted in enhanced chemosensitivity, whereas NOTCH1 and mTOR inhibition did not display objective response^45^. In addition, combination of GSIs with gemcitabine showed partial response or stable disease of phase I clinical trial^46^. Consequently, not all GSIs combined with chemotherapy have added benefit, and optimal regimen for maximal tumor reduction and minimal side effect required further exploration^44^. Given these diverse conditions, a thorough understanding of the genetic, transcriptomic, and proteomic signatures is imperative before initiating treatment.

Given the large number of disease settings in which aberrant NOTCH1 signaling is involved, improved understanding of the NOTCH1 pathway and rational target strategy is a pressing need^47^. We provided evidence that modulating NOTCH1 signaling pathway may be of great potential to eliminate CSC populations in HNSCC with a rather tolerable γ-secretase inhibitor^48^. The fact that combinatorial regimen of NOTCH inhibition and chemotherapy synergistically attenuates chemotherapy-enriched CSC population opens up interesting possibilities for combining NOTCH1 blockade with conventional therapeutic strategies, which provides a rationale for further clinical trials.

## Materials and Methods

Detailed methods and procedures are provided in the Supplementary Appendix files.

### Cell culture, *in vitro* tumor sphere formation assay

HNSCC cell lines CAL27 and FaDu were purchased from the American Type Culture Collection (ATCC, Manassas, VA). Cell lines were maintained in Dulbecco’s modified Eagle’s medium (DMEM)/F12, 10% fetal bovine serum (FBS), at 5% CO_2_ and 37 °C humidified incubators with anti-vibration platform. For the tumor sphere culture assay, single-cell suspensions were resuspended in culture media containing 1% N2 supplement (Gibco), 2& B27supplement, 20 ng/mL basic fibroblast growth factor (bFGF-2, R&D), and 10 ng/mL epithelial growth factor (EGF, R&D) and plated in ultra-low attachment plates (Corning) at a density of 1 × 10^3^ cells per well as previously reported^49^. Medium was replenished twice a week and spheres counted within 2 weeks. To evaluate the tumor sphere size, cells were plated in 96-well ultra-low attachment plates (Corning), at a density of 1 cell per well. The number and size of spheres formed were evaluated using an inverted microscope.

### RNA interference and cell viability

RNA interference was performed as previous described^49^. Briefly, CAL 27 and FaDu cells were seeded in 6 cm culture dishes and allowed to grown to 80% confluence, transfected with non-targeting negative control siRNA (Qiagen, Valencia, CA), NOTCH1 siRNA (Hs_NOTCH1_1 FlexiTube siRNA and Hs_NOTCH1_2 FlexiTube siRNA) with Hiperfect transfection reagent (Qiagen) according to the manufacturer’s instruction^50^. The knock down efficiency with NOTCH1 protein at an indicated time (24 h) were confirmed by western blot. MTS assay (Promega, Madison, WI) of DAPT was performed according to the protocol suggested by the manufacturer^49^. The percentage of cell growth was calculated based on 100% growth at 24 h after transfection.

### Drugs, nude mice and DAPT treatment

The γ-Secretase inhibitor DAPT (N-[N-(3,5-Difluorophenacetyl-L-alanyl)]-S-phenylglycine t-Butyl Ester, GSI-IX) purchased from Selleck Chemicals (Houston, TX, USA) was initially dissolved in 100% ethanol at a concentration of 20 mg/ml, and stored at −20 °C. The working solution was further diluted in PBS with a final concentration of 2 mg/ml, immediately before use. The PBS as a vehicle was used as a negative control. Female BALB/c nude mice (18–20 g) with 6–8 weeks of age were housing in the Experimental Animal Center of Wuhan University in pressurized ventilated cages according to institutional regulations. All proposals were approved and supervised by the institutional animal care and use committee of Wuhan University. All animal studies were conducted in accordance with the NIH guidelines for the Care and Use of Laboratory Animals. For *in vitro* experiment, the cells were treated with 5 μM and 10 μM DAPT at indicated time. For nude mouse xenograft chemotherapeutic experiment, human HNSCC cell line CAL27 or SCC9 were injected subcutaneously in nude mice. Two weeks after injection, the tumor was visible. The mice were randomly divide into control group (PBS, i.p. daily, n = 10 mice), 10 mg/kg DAPT treated group (i.p. daily, n = 10 mice) and 20 mg/kg DAPT treated group (i.p. daily, n = 10 mice) and were observed for fourteen days. For combined DAPT and chemotherapy experiments, cisplatin (CDDP, 10 mg/kg, 1 dose at day18), docetaxel (DTX, 10 mg/kg, 1 dose at day18) and 5-fluorouracil (5-FU, 15 mg/kg) with or without additional 10 mg/kg DAPT were infused intraperitoneally with vehicle only as a control (n = 5 mice respectively).

### Flow cytometry and side population

Flow cytometry staining was performed as previously described^49^. The cells were analyzed on a FACS caliber flow cytometer equipped with Cell Quest software, and gated by the side scatter and forward scatter filters (Becton Dickinson, Mountain View, CA). Side population discrimination was based on Goodell^51^ and colleagues, with detailed procedure in supplementary materials.

### Western blot

The Western blot analysis was performed according to our previous procedures^49^. Briefly, about 40 ug of protein from each sample were denatured and then loaded in each lane of NuPAGE 4–12% Bis-Tris precast gel. Subsequently, proteins were transferred onto a membrane and blocked for 1 hour, then incubated with primary antibodies overnight, finally by incubation with horseradish peroxidase-conjugated secondary antibody (Pierce, Rockford, IL). The following primary antibody dilutions were used: 1:1000 for NOTCH1, HES1, SOX2, ALDH1, BMI1, and CD44.

### Quantitative real-time PCR

Quantitative real-time PCR was performed as previous described. Briefly, miRNeasy Mini kit (Qiagen, Hilden, Germany) was used to extract the total RNA from DAPT treated CAL27 and FaDu cells. RNA quality control was performed as previously described^50^. Human *HES1, ALDH1A1, BMI1 and SOX2* primer was purchased from Qiagen. The mRNAs were subjected to quantitative 2-step real-time PCR using transcript III kit (Invitrogen) and SYBR green PCR kit (Bio-Rad). qRT-PCR was performed in triplicate on the Chrom 4 real-time PCR System (Bio-Rad, Hercules, CA) using a GAPDH probe as an internal control, according to the protocol suggested by the manufacturer.

### Human HNSCC tissue microarray

The custom made HNSCC tissue microarrays of humans used in this study were described previously^49^, with the informed constent obtained from all subject and approval of the Medical Ethics Committee of School and Hospital of Stomatology, Wuhan University. Methods were carried out in accordance with the approved guidelines. These tissue microarray slides included 59 confirmed cases of HNSCC, 39 normal oral mucosa and 13 oral epithelial dysplasia, 5 paired lymph node metastasis, 5 recurrences after radiotherapy and 12 TPF chemotherapy. The 12 HNSCC patients receive 2 round combined CDDP, DTX, and 5-FU therapy with the same protocol of Zhang’s clinical trial^40^. Patient samples with both biopsy and surgical specimen after 2 rounds were involved in these custom-made tissue microarrays.

### Histology and immunochemistry

The following antibodies were used in this study: NOTCH1, HES1, SOX2, (Cell Signaling Technology), ALDH1, CD44, CD133 (Proteintech Group). All antibodies were used for IHC at a dilution of 1:1000 or 1:200, respectively. Tumors and control tissues such as normal mucosa, dysplasia was carefully dissected from the mice and fixed in 10% buffered formalin overnight. Immunohistochemistry using ABC kit was performed as previous described^49^ and briefly described in Supplementary Appendix material and methods.

### Statistical analysis

Statistical data analysis was performed with GraphPad Prism 5.03 (GraphPad Software, Inc., La Jolla, CA) statistical packages. The differences in immunostaining and protein levels among each group were analyzed by the One-way ANOVA followed by the post-Tukey or Bonferroni multiple comparison tests. The Student *t* test was used to evaluate differences in the total tumor area of the mice treated with DAPT and control group. Two-tailed Pearson statistics were used for correlated expression of these markers after confirmation of the sample with Gaussian distribution. All data were presented as mean ± SEM, statistical significance was defined as the p-value was <0.05.

## Additional Information

**How to cite this article**: Zhao, Z.-L. *et al.* NOTCH1 inhibition enhances the efficacy of conventional chemotherapeutic agents by targeting head neck cancer stem cell. *Sci. Rep.* **6**, 24704; doi: 10.1038/srep24704 (2016).

## Supplementary Material

Supplementary Information
