# Supplementary material for: NOTCH1 inhibition enhances the efficacy of conventional chemotherapeutic agents by targeting head neck cancer stem cell
Source: Sci Rep. 2016 Apr 25;6:24704. doi: 10.1038/srep24704 (PMC4842967; doi:10.1038/srep24704)
Supplement: Supplementary Information [file srep24704-s1.doc]

**Supplementary Information**

**NOTCH1 inhibition enhances the efficacy of conventional chemotherapeutic agents by targeting head neck cancer stem cell**

Zhi-Li Zhao^a,1^, Lu Zhang^a,1^,Cong-Fa Huang^a^, Si-Rui Ma^a^,Lin-Lin Bu^a^, Jian-Feng Liu^a^, Guang-Tao Yu^a^, Bing Liu^b^, J. Silvio Gutkind^c^, Ashok B. Kulkarni^d^, Wen-Feng Zhang^b^, Zhi-Jun Sun^a,b,d^,*

^a^*The State Key Laboratory Breeding Base of Basic Science of Stomatology & Key Laboratory of Oral Biomedicine, Ministry of Education;*

^b^*Department of Oral Maxillofacial-Head Neck Oncology, School and Hospital of Stomatology, Wuhan University, Wuhan, China;*

^c^*Oral and Pharyngeal Cancer Branch, National Institute of Dental and Craniofacial Research, National Institutes of Health, Bethesda, MD USA;*

^d^*Functional Genomics Section, Laboratory of Cell and Developmental Biology, National Institute of Dental and Craniofacial Research, National Institutes of Health, Bethesda, MD USA.*

^1^Z.L.Zhao and L.Zhang contributed equally to this work.

^*^Corresponding author. Address: School and Hospital of Stomatology, Wuhan University, 237 Luoyu Road, Wuhan, 430079, China. E-mail: zhijundejia@163.com

**Supplementary Materials and Methods**

**Cell culture, tumor sphere formation assay.** HNSCC cell lines CAL27 and FaDu were purchased from the American Type Culture Collection (ATCC, Manassas, VA). Cell lines were maintained in Dulbecco’s modified Eagle’s medium (DMEM)/F12, 10% fetal bovine serum (FBS), at 5% CO2 and 37℃ humidified incubator with anti-vibration platform. For the tumor sphere culture assay, single-cell suspensions were resuspended in culture media containing 1% N2 supplement (Gibco), 20 ng/mL basic fibroblast growth factor (bFGF-2, R&D), and 10 ng/mL epithelial growth factor (EGF, R&D) and plated in ultra-low attachment plates (Corning) at a density of 1 × 10^3^  cells per well. Medium was replenished twice a week and spheres counted within 2 weeks. The number and size of spheres formed were evaluated using an inverted microscope.

**Flow cytometry and side population.** Flow cytometry staining was performed as described previously. Briefly, Single cell suspensions were made by trypsinizing cultured cells or gently homogenizing xenograft tissue from followed by Dispase enzyme digestion (Roche). For tumor tissues, we followed a published protocol[^1^](#_ENREF_1). These cells were labeled with fluorescence-conjugated antibodies (BD Pharmingen) and isotype-matched IgG controls. The cells were analyzed on a FACS caliber flow cytometer equipped with Cell Quest software, and gated by the side scatter and forward scatter filters (Becton Dickinson, Mountain View, CA). Side population discrimination was based on Goodell[^2-4^](#_ENREF_2) and colleagues with slight adaptation according to the recommendation of Simone and colleagues[^5^](#_ENREF_5). Briefly, cells (1 × 10^6^ cells/mL) were resuspended in prewarmed DMEM (Life Technologies) with 2% FBS (Life Technologies) containing freshly added Hoechst 33342 (5 μg/mL final concentration) for 90 minutes at 37°C in water bath with intermittent mixing and darkness, either alone or in the presence of 50 μmol/L verapamil (Sigma). At the end of incubation, samples were chilled on ice, centrifuged down at 4°C and resuspended in ice-cold PBS with 2% FBS(Life Technologies). 7-AAD at a final concentration of 2μg/mL was added for 5 minutes before fluorescence-activated cell sorting (FACS) analysis, which allows for the discrimination of dead versus live cells. To dissociate multicellular aggregates, the cells were filtered with 40-μm cell strainer. The Hoechst33342 dye was excited with the UV laser at 355 nm and its fluorescence was dual-wavelength analyzed (blue, 450/65 nm; red, 670/30 nm) with FACSVantage SE (Becton Dickinson).

**Western blot.** The Western blot analysis was performed according to our previous procedures. Briefly, about 40ug of protein from each sample were denatured and then loaded in each lane of NuPAGE 4-12% Bis-Tris precast gel. Subsequently, proteins were transferred onto a membrane and blocked for 1 hour, then incubated with primary antibodies overnight, finally by incubation with horseradish peroxidase-conjugated secondary antibody (Pierce, Rockford, IL). The following primary antibody dilutions were used: 1:1000 for Notch1, SOX2, ALDH1, CD44, and CD133.

**Histology and Immunochemistry**. The following antibodies were used in this study: Notch1, HES1, SOX2, (Cell Signaling Technology), ALDH1, CD44, CD133 (Proteintech Group, Chichago, IL). All antibodies were used for IHC at a dilution of 1:1000 or 1:200, respectively. Tumors and control tissues such as normal mucosa, dysplasia was carefully dissected from the mice and fixed in 10% buffered formalin overnight. Immunohistochemistry using ABC kit was performed as previous described and brief described in supplementary material and methods.

**Supplementary Figure and Figure legends**


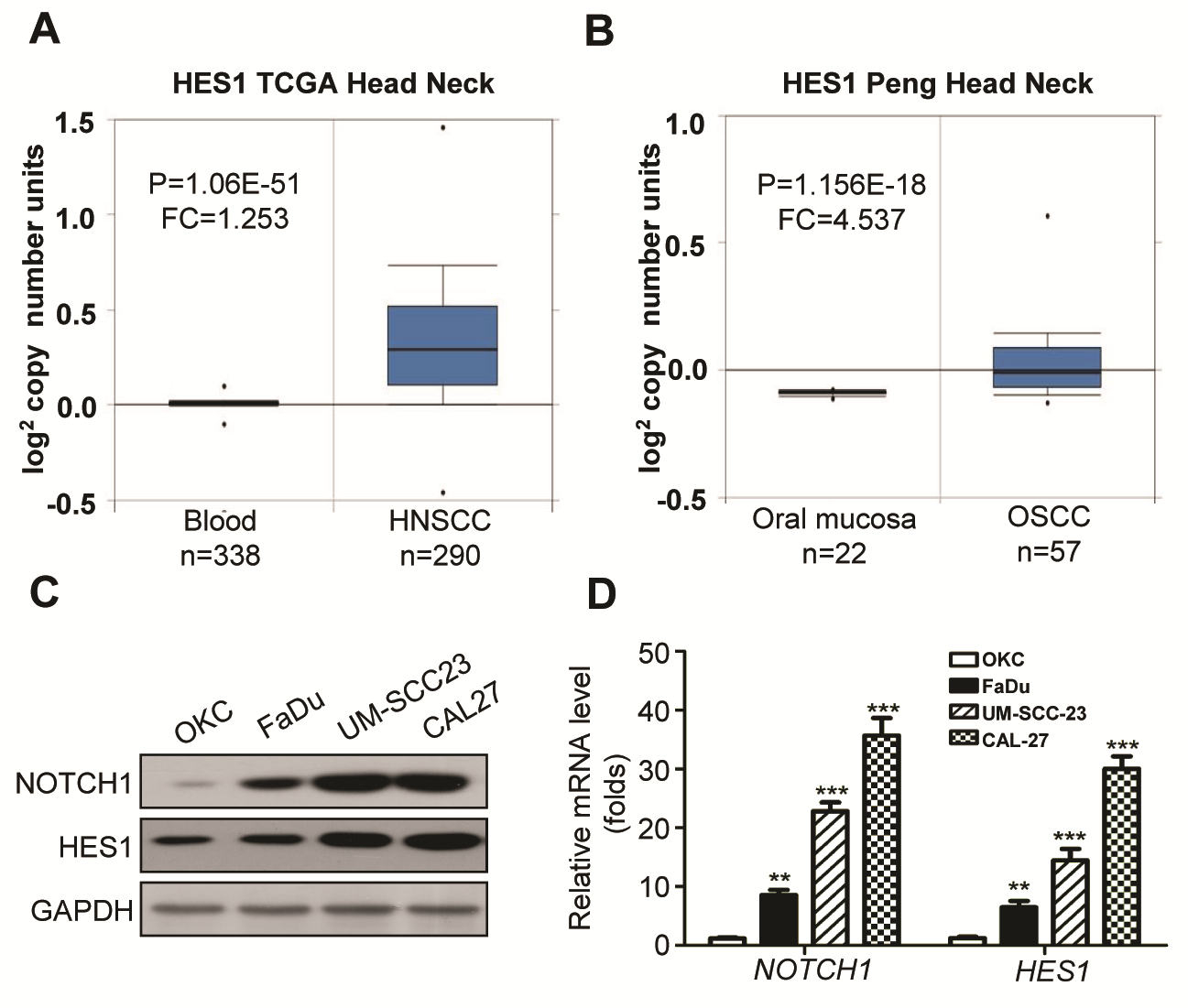


**Figure S1.** Increased *HES1* DNA copy number and mRNA in head neck cancer. (A) Downstream target of Notch1- *HES1* DNA copy number from TCGA head neck cancer dataset and (B) mRNA expression from Peng’s dataset (as log2 median –centered ratio) for head neck cancer versus normal counterpart shown as box whisker data. Data retrieve from ONCOMINE database. (C) Representative Western blotting shows increased NOTCH1 and HES1 expression in oral keratinocyte (OKC) and head neck squamous cell carcinoma (HNSCC) lines FaDu, UM-SCC23 and CAL27. GAPDH was used as loading control. Experiment was repeated twice. (D) Significant increased mRNA levels of NOTCH1 and HES1 in HNSCC cell lines as compared with OKC. The relative expression level was calculated using the ΔΔCt method and normalized to primary cultured oral keratinocyte (OKC).


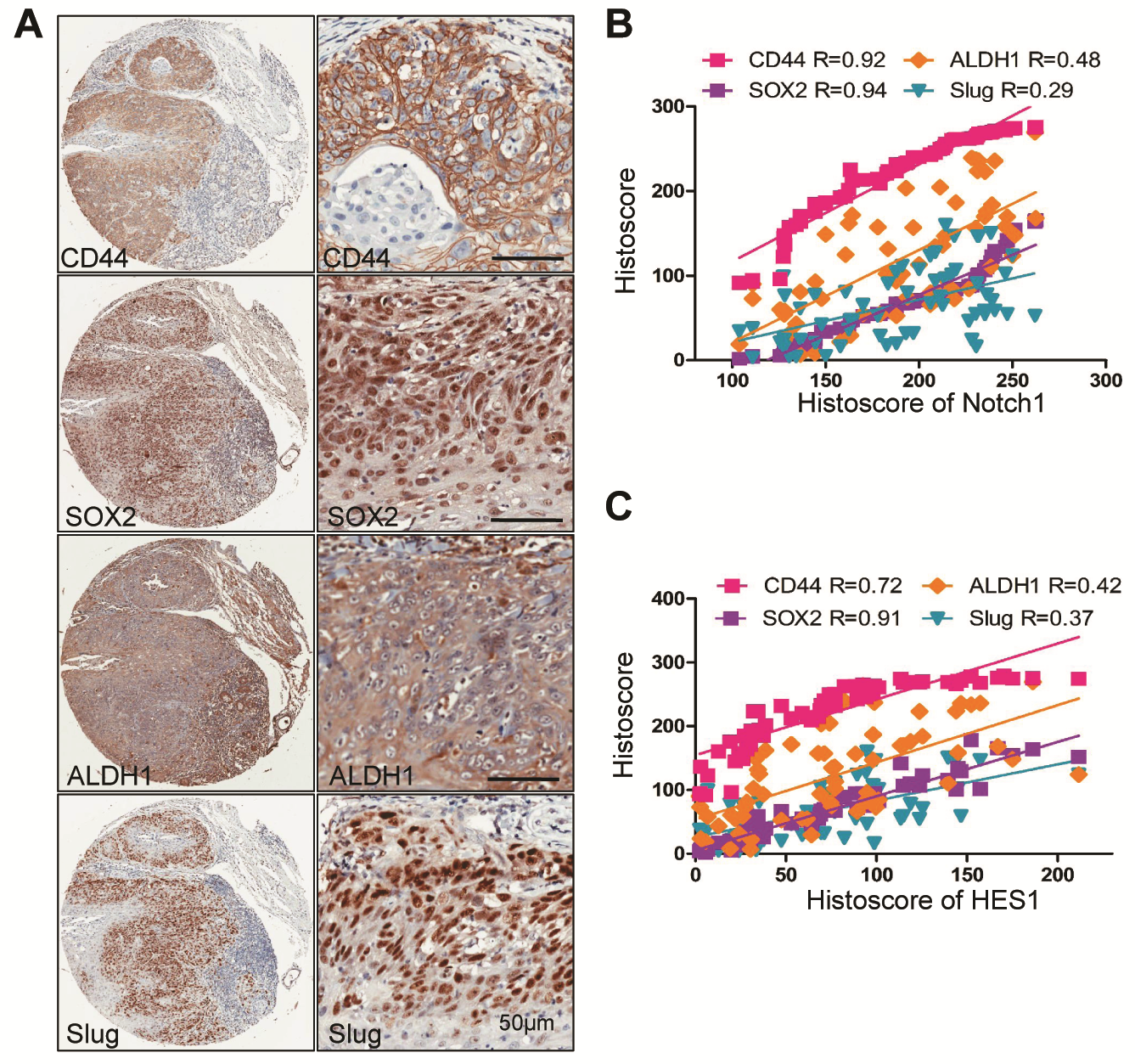


**Figure S2.** Increased NOTCH signaling correlates with cancer stem cells markers.(A) Representative immunohistochemical staining of cancer stem cells markers CD44, SOX2, ALDH1, and Slug expression were increased in human HNSCC; Scale bar, 50μm. (B) NOTCH1 expression was correlated with CD44 (R=0.92, *P*<0.05), ALDH1 (R=0.48, *P*<0.05), SOX2 (R=0.94, *P*<0.05), Slug (R=0.29, *P* <0.05) in HNSCC determined by Pearson correlation analysis. Histoscore based on Aperio quantification and statistics with GraphPad prism, two-tail Pearson correlation. (C) HES1 was correlated with CD44 (R=0.72, *P* <0.05), ALDH1 (R=0.42, *P* <0.05), SOX2 (R=0.91, *P* <0.05), Slug (R=0.37, *P* <0.05) in HNSCC.

**
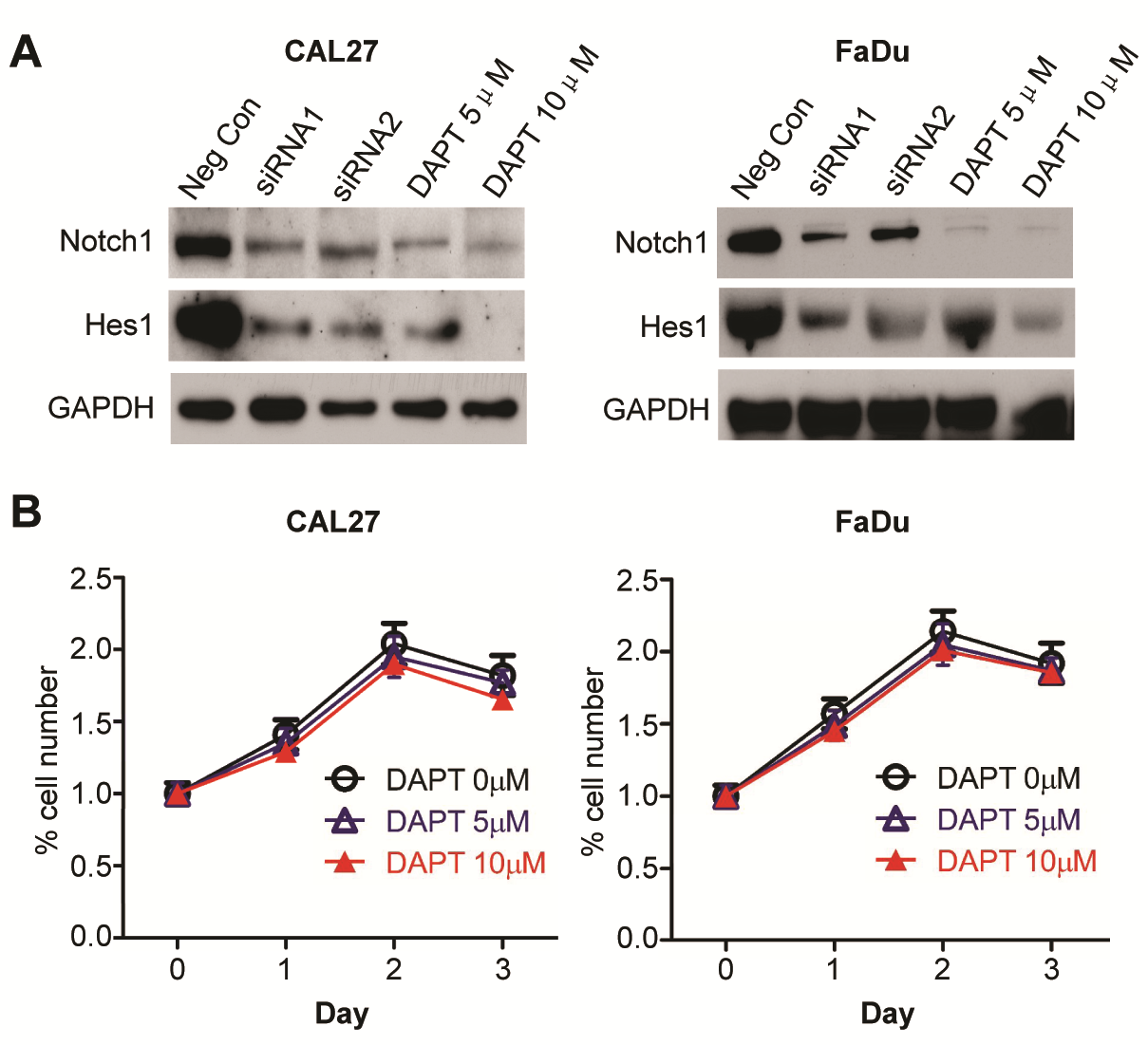
**

**Figure S3**. Inhibition efficacy of NOTCH1 siRNA and cell viability of DAPT in human HNSCC cell line CAL27 and FaDu. (A) Representative Western blot images of negative control (Mock), NOTCH1 siRNA_1, NOTCH1 siRNA_2, 5μM and 10 μM DAPT after 24h transfection or treatment. (B) Cell viability present as relative cell number with 5μM and 10 μM DAPT treatment at indicated time (*P*>0.05).


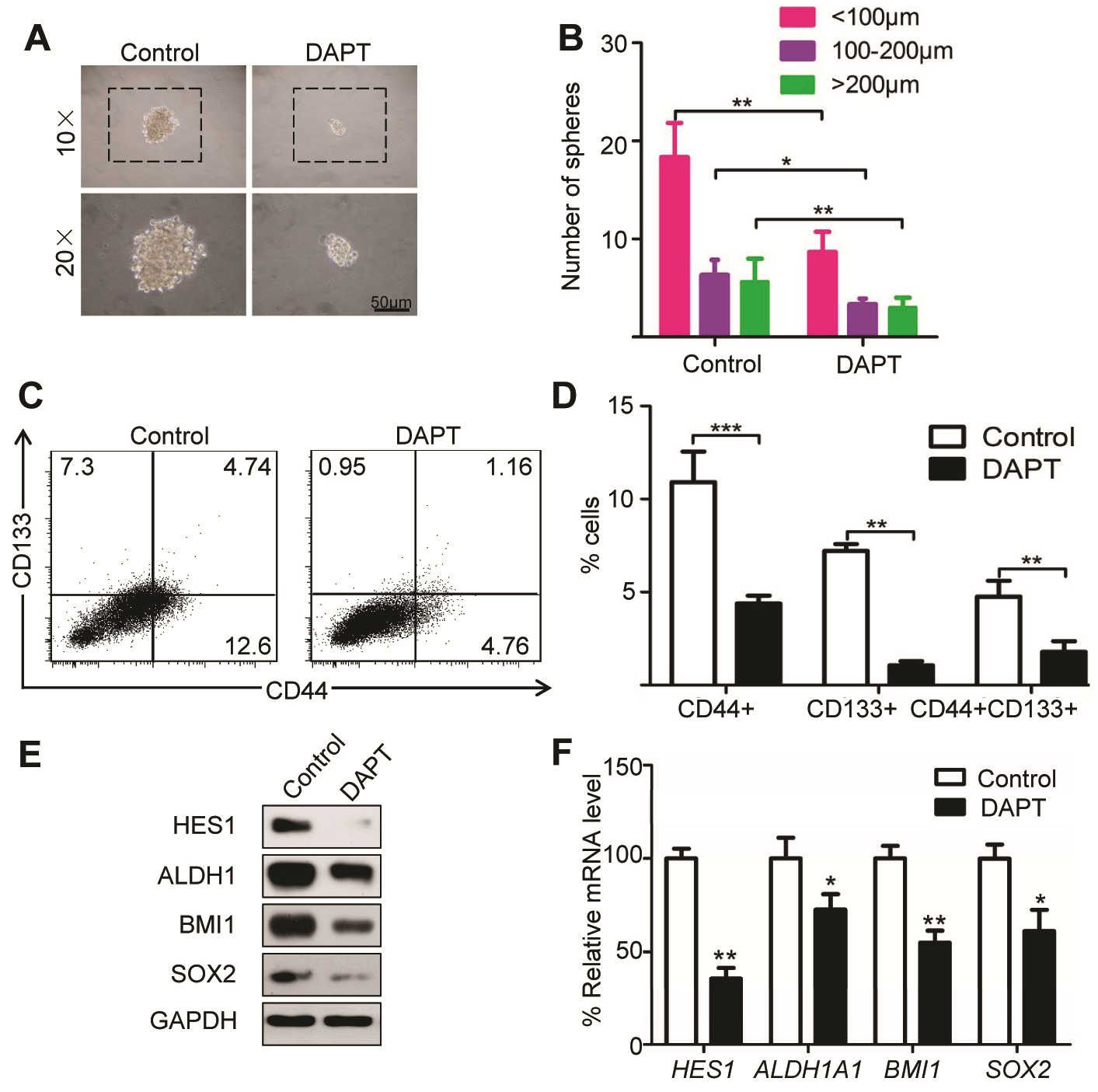


**Figure S4**. NOTCH inhibition attenuates cancer stem cells phenotype of FaDu cell line. (A) Representative images of FaDu *in vitro* tumor sphere formation assay after 14days culture in the serum free media with or without 10μM DAPT; Scale bar, 50μm. Experiment was repeated twice. (B) Quantification of the number and size of FaDu tumor spheres with or without DAPT treatment; Data presented as mean±SEM, *, *P*<0.05; **, *P*<0.01. (C) Representative flow cytometry and quantification (D) indicate decrease in CD44+CD133+ and CD44+CD133- cell population by DAPT treatment FaDu cell line; Experiment was repeated twice. **, *P*<0.01; **, *P*<0.01. (E) Western blotting analysis shows DAPT decrease ALDH1, BMI1 and SOX2 expression in FaDu cell line. (F) The mRNA levels of *HES1*, *ALDH1A1*, *BMI1* and *SOX2* were analyzed with qPCR in FaDu cell line; data presented as normalized relative mRNA level, *, *P* <0.05; **, *P* <0.01.The experiment was repeated twice in triplicate.

**
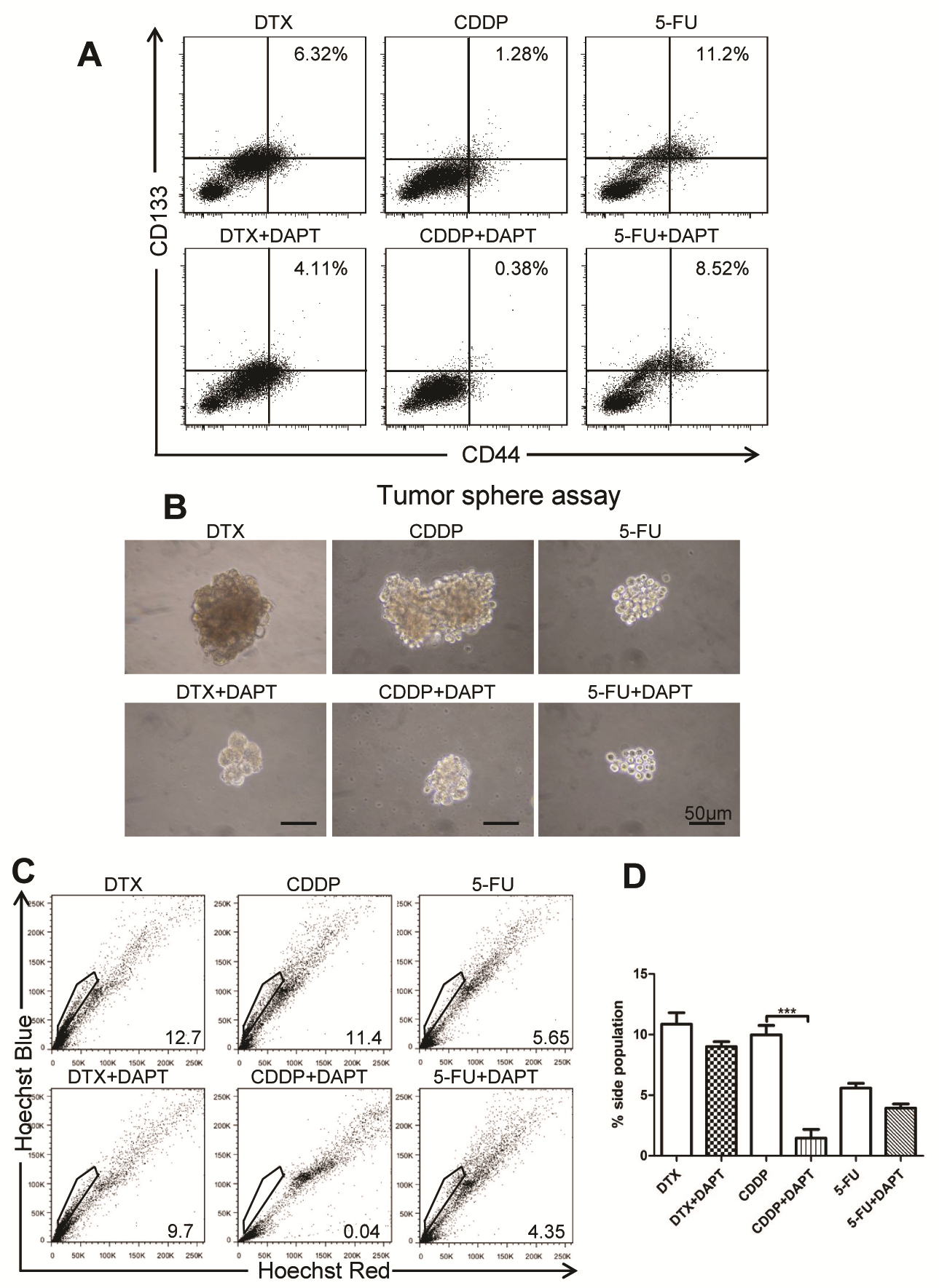
**

**Figure S5.** Suppression of NOTCH signaling inhibits chemoresistance of FaDu cell line. (A) Representative flow cytometry photos shows DAPT treatment attenuates chemotherapy agent Docetaxel (DTX, 10μM), cisplatin (CDDP, 10μM), 5-fluorouracil (5-FU,15μM) induced CD44+CD133+ CAL27 cell population. Experiment was repeated twice. (B)Representative tumor sphere formation of combined DAPT treatment with chemotherapeutic agents; Scale bar, 50μm. (C) Representative flow cytometry images show side population changes of DTX, CDDP, 5-FU treated CAL27 cells with or without DAPT. Stained with Hoechst 33342 and excited by UV light; Hoechst Red, 675nm channel; Hoechst Blue, 450nm channel. (D) Quantification of side populations from triple experiments; Data presented as mean±SEM, ***, *P* <0.001. The experiment was repeated twice in triplicate.

**Supplementary References**

1 Ma, S. R., Wang, W. M., Huang, C. F., Zhang, W. F. & Sun, Z. J. Anterior gradient protein 2 expression in high grade head and neck squamous cell carcinoma correlated with cancer stem cell and epithelial mesenchymal transition. *Oncotarget* **6**, (2015).

2 Goodell, M. A., Brose, K., Paradis, G., Conner, A. S. & Mulligan, R. C. Isolation and functional properties of murine hematopoietic stem cells that are replicating in vivo. *J Exp Med* **183**, (1996).

3 Camargo, F. D., Chambers, S. M., Drew, E., McNagny, K. M. & Goodell, M. A. Hematopoietic stem cells do not engraft with absolute efficiencies. *Blood* **107**, (2006).

4 Challen, G. A., Boles, N. C., Chambers, S. M. & Goodell, M. A. Distinct hematopoietic stem cell subtypes are differentially regulated by TGF-beta1. *Cell Stem Cell* **6**, (2010).

5 Golebiewska, A., Brons, N. H., Bjerkvig, R. & Niclou, S. P. Critical appraisal of the side population assay in stem cell and cancer stem cell research. *Cell Stem Cell* **8**, (2011).
